# How to assess success of treatment when using multiple doses: the case of misoprostol for medical abortion

**DOI:** 10.1186/s13063-015-1035-0

**Published:** 2015-11-07

**Authors:** Armando H. Seuc, Iqbal H. Shah, Moazzam Ali, Claudia Diaz-Olavarrieta, Marleen Temmerman

**Affiliations:** Department of Reproductive Health and Research, World Health Organization, 1211 Geneva 27, Switzerland; Department of Global Health and Population, Harvard T. H. Chan School of Public Health, 665 Huntington Avenue, Boston, MA 02115 USA; Population Council Mexico, Insurgentes Sur 2453 – Torre Murano Piso 9 Local 903, Col. Tizapán, Del. Álvaro Obregón, México City, 01090 Mexico

**Keywords:** Medical abortion, Binomial proportion, Survival analysis, Competing risks

## Abstract

**Background:**

The assessment of treatment success in clinical trials when multiple (repeated) doses (courses) are involved is quite common, for example, in the case of infertility treatment with assisted reproductive technology (ART), and medical abortion using misoprostol alone or in combination with mifepristone. Under these or similar circumstances, most researchers assess success using binomial proportions after a certain number of consecutive doses, and some have used survival analysis. In this paper we discuss the main problems in using binomial proportions to summarize (the overall) efficacy after two or more consecutive doses of the relevant treatment, particularly for the case of misoprostol in medical abortion studies. We later discuss why the survival analysis is best suited under these circumstances, and illustrate this by using simulated data.

**Methods:**

The formulas required for the binomial proportion and survival analysis (without and with competing risks) approaches are summarized and analytically compared. Additionally, numerical results are computed and compared between the two approaches, for several theoretical scenarios.

**Results:**

The main conceptual limitations of the binomial proportion approach are identified and discussed, caused mainly by the presence of censoring and competing risks, and it is demonstrated how survival analysis can solve these problems. In general, the binomial proportion approach tends to underestimate the “real” success rate, and tends to overestimate the corresponding standard error.

**Conclusions:**

Depending on the rates of censored observations or competing events between repeated doses of the treatment, the bias of the binomial proportion approach as compared to the survival analysis approaches varies; however, the use of the binomial approach is unjustified as the survival analysis options are well known and available in multiple statistical packages. Our conclusions also apply to other situations where success is estimated after multiple (repeated) doses (courses) of the treatment.

## Background

The World Health Organization (WHO) indicates that for pregnancies of gestational age between 9 and 12 weeks (63–84 days), the recommended method for medical abortion is 200 mg mifepristone administered orally followed 36 to 48 hours later by 800 μg misoprostol administered vaginally. Subsequent misoprostol doses should be 400 μg, administered either vaginally or sublingually, every 3 hours up to a maximum of four further doses. For pregnancies of gestational age over 12 weeks (84 days), the recommended method for medical abortion is 200 mg mifepristone administered orally followed 36 to 48 hours later by an initial dose of 400 μg orally or 800 μg vaginally, with further doses of 400 μg misoprostol administered either vaginally or sublingually every three hours, up to four further doses [1].

Under these or similar circumstances, many papers assess the efficacy of multiple doses of misoprostol calculating the binomial proportion of successful abortions (without surgical intervention) after a certain number of consecutive doses (or courses), usually two [2–8]; see also studies included in the systematic review [9]. Other studies also use formal survival analysis of “induction to conception expulsion” time, in hours [10, 11].

To the best of our knowledge, no paper has attempted to discuss or question the statistical approaches to be applied under these circumstances, the exception being Gallo and co-authors [9]. These authors did not address the topic in detail (just one paragraph within the “outcome measures” section) but make two main points; first they argue that the efficacy of a second dose of misoprostol should not be estimated from those who fail with the first dose and go for the second dose, because of the (obvious) attrition bias [12] that would render inappropriate any comparisons between success rates of the second versus the first dose. Though this is correct, we have not seen any studies comparing the efficacy across doses (such as second versus first or third versus first).

Secondly, the authors argue that the use of binomial proportions to summarize the success of several consecutive doses of misoprostol (the combined or overall success rate) ignores important information that is accounted for when survival analysis techniques are applied. This is undoubtedly the case, but we need to specify the additional information that is provided, how to use it, and the eventual impact on the final results.

In this paper we present the main problems encountered when using binomial proportions to summarize (the overall) efficacy after administration of two or more consecutive doses of misoprostol in medical abortion studies. We later discuss why the competing risk survival analysis is best suited under these circumstances, and illustrate this by using simulated data. Finally we present some concluding remarks. In this paper “successful abortion” stands for “complete expulsion of the products of conception without surgical intervention”.

## Methods

We present and compare four statistical approaches for assessing success when using multiple doses, the binomial proportion approach and three survival analysis approaches: Kaplan-Meier, Life Table, and the (Competing Risk) Cumulative Incidence rate.

At first the approaches are analytically compared in terms of biases in point estimations and corresponding standard errors. Additionally, 18 artificially created scenarios are generated combining three levels for the rate of the main event with the first dose, two levels for the rate of the main event with the second dose, and three levels for the rate of the competing risk event and censoring, and the approaches are then numerically compared in Table 1.

**Table 1:** Estimation of the success rate of medical abortion after two doses of misoprostol under different scenarios for the rates of the main event medical abortion, the competing event surgical abortion, and censored data ({1}–{8}), using the binomial approach ({9}, {14}), the Kaplan-Meier (KM) approach ({10}, {15}), the Life Table (LT) approach ({11}, {16}), and the competing risk survival approach ({12}, {13}, {17}, {18})

Rate of main event with 1st dose	Rate of main event with 2nd dose (^a^)	Rate of competing event and censoring (^b^)	Assumptions for number of main events (medical abortions (r1, r2), competing events (d1, d2), and censored observations (c1, c2)).								POINT ESTIMATION					STANDARD ERROR				
			{1}	{2}	{3}	{4}	{5}	{6}	{7}	{8}	{9}	{10}	{11}	{12}	{13}	{14}	{15}	{16}	{17}	{18}
			n1	r1	d1	c1	n2	r2	d2	c2	bin	KM^c^	LT^c^	CI1(2)	CI2(2)	bin	KM^c^	LT^c^	CI1(2)	CI2(2)
Low (70 %)	Low (62 %)	Low	1000	700	15	14	271	168	102	1	86.79 %	88.60 %	93.20 %	87.67 %	12.23 %	1.07 %	1.04 %	0.90 %	1.03 %	1.03 %
		Medium	1000	700	30	27	243	151	91	1	85.07 %	88.64 %	93.47 %	86.78 %	13.11 %	1.13 %	1.08 %	0.91 %	1.06 %	1.06 %
		High	1000	700	60	48	192	119	72	1	81.90 %	88.59 %	93.90 %	84.87 %	15.00 %	1.22 %	1.19 %	0.95 %	1.11 %	1.11 %
	High (66 %)	Low	1000	700	15	14	271	179	91	1	87.89 %	89.82 %	94.08 %	88.82 %	11.07 %	1.03 %	0.99 %	0.83 %	0.99 %	0.99 %
		Medium	1000	700	30	27	243	160	82	1	86.04 %	89.75 %	94.24 %	87.78 %	12.11 %	1.10 %	1.04 %	0.85 %	1.02 %	1.02 %
		High	1000	700	60	48	192	127	65	0	82.67 %	89.84 %	94.70 %	85.87 %	14.12 %	1.20 %	1.14 %	0.88 %	1.08 %	1.08 %
Medium (80 %)	Low (70 %)	Low	1000	800	10	10	180	126	53	1	92.60 %	94.00 %	96.61 %	93.30 %	6.59 %	0.83 %	0.78 %	0.63 %	0.78 %	0.78 %
		Medium	1000	800	20	18	162	113	48	1	91.34 %	93.95 %	96.71 %	92.56 %	7.33 %	0.89 %	0.82 %	0.64 %	0.82 %	0.81 %
		High	1000	800	40	32	128	90	38	0	88.96 %	94.06 %	97.03 %	91.25 %	8.75 %	0.99 %	0.89 %	0.65 %	0.87 %	0.87 %
	High (75 %)	Low	1000	800	10	10	180	135	45	0	93.50 %	95.00 %	97.26 %	94.25 %	5.75 %	0.78 %	0.72 %	0.56 %	0.73 %	0.73 %
		Medium	1000	800	20	18	162	122	40	0	92.15 %	95.03 %	97.39 %	93.45 %	6.55 %	0.85 %	0.75 %	0.57 %	0.77 %	0.77 %
		High	1000	800	40	32	128	96	32	0	89.60 %	95.00 %	97.57 %	92.00 %	8.00 %	0.97 %	0.83 %	0.59 %	0.83 %	0.83 %
High (90 %)	Low (79 %)	Low	1000	900	5	5	90	71	19	0	97.13 %	97.89 %	98.87 %	97.49 %	2.51 %	0.53 %	0.47 %	0.36 %	0.49 %	0.49 %
		Medium	1000	900	10	9	81	64	17	0	96.40 %	97.90 %	98.93 %	97.11 %	2.89 %	0.59 %	0.49 %	0.36 %	0.52 %	0.52 %
		High	1000	900	20	16	64	51	13	0	95.06 %	97.97 %	99.06 %	96.37 %	3.62 %	0.69 %	0.54 %	0.36 %	0.57 %	0.57 %
	High (85 %)	Low	1000	900	5	5	90	77	13	0	97.65 %	98.56 %	99.26 %	98.13 %	1.87 %	0.48 %	0.40 %	0.29 %	0.42 %	0.42 %
		Medium	1000	900	10	9	81	69	12	0	96.89 %	98.52 %	99.27 %	97.67 %	2.33 %	0.55 %	0.42 %	0.30 %	0.47 %	0.47 %
		High	1000	900	20	16	64	54	10	0	95.44 %	98.44 %	99.29 %	96.75 %	3.25 %	0.66 %	0.48 %	0.31 %	0.54 %	0.54 %

{1} n1: subjects receiving the 1st dose

{2} r1: number of successes after the 1st dose (and before the 2nd)

{3} d1: number of competing events after 1st dose (and before 2nd)

{4} c1: number of censored data between the 1st and the 2nd doses

{5} n2: subjects receiving the 2nd dose

{6} r2: number of successes after the 2nd dose (and before end of study)

{7} d2: number of competing events after 2nd dose (and before end of study)

{8} c2: number of censored data between the 2nd dose and end of study

{9}: success rate using the binomial proportion

{10}: success rate using KM^c^

{11}: success rate using Life Table with actuarial assumptions^c^

{12}: success rate using competing risk approach (cmprsk package in R)

{13}: as in {17} but for the competing event “surgical abortion”

{14}: standard error of point estimation in {13}

{15}: standard error of point estimation in {14}

{16}: standard error of point estimation in {16}

{17}: standard error of point estimation in {17}

{18}: standard error of point estimation in {18}

^a^: rate of event with second dose was assumed lower than with first dose, “low” (12 % relative reduction) and “high” (6 % relative reduction)

^b^: For j = 1, as a fraction of (n1-r1), or (n1-r1-d1): low = 5 %, medium = 10 %, high = 20 %; for the second dose, c2 = 0.01*(n2-r2), and d2 = n2-r2-c2

^c^: All events occur exactly at times (doses) 1 and 2. Life Table and KM rates were computed with SPSS, and Cumulative Incidence (CI) rates with cmprsk package in R; for this purpose 18 datasets were created replicating the assumptions in columns {1} to {8}, and the corresponding packages were applied

The three levels assumed for the rate of the main event with the first dose were “low 70 %”, “medium 80 %”, and “high 90 %”; the two levels assumed for the rates of the main event with the second dose were “low” and “high” corresponding to 12 % and 6 % relative reduction with respect to the first dose’s rate. The three levels assumed for the rates of the competing event and censoring with the first dose were “low”, “medium”, and “high” corresponding to 5 %, 10 %, and 20 % of all remaining units (columns {3} and {4} in Table 1). For the second dose we assumed an almost zero rate (1 %) of censoring and all other remaining subjects were reconverted into the competing risk event (surgical abortion), which is the usual practice in these types of studies (see columns {7} and {8} in Table 1).

The figures in columns {2} to {8} in Table 1 are derived from the assumed rates in the 18 different scenarios (rows), and by making the initial sample size equal to 1,000 (=n1, column {1} in Table 1) in all scenarios. Figures in columns {9} to {13} provide the point estimations for the four statistical approaches, and figures in columns {14} to {18} provide the corresponding standard errors, using the formulas presented in the Results sections. For the competing risk approach results are presented both for the rate of the main event and the rate of the competing event, point estimations (columns {12} and {13}), and corresponding standard errors (columns {17} and {18}).

## Results

### The problem of using binomial proportions

A binomial proportion is derived from a random variable *X* that summarizes the result of “n” independent repetitions of a Bernoulli experiment, each with probability “*P*” of success, by providing the total number of successes (or the proportion of successes) out of “n” repetitions; the binomial distribution is characterized by two parameters, “n” and “*P*” and is denoted as *B*(*n*,*P*). The probability that *X* takes the value (number of successes) “r” is given by [13]:

$$ P\left(X=r\right) = \left(\begin{array}{c}\hfill n\hfill \\ {}\hfill r\hfill \end{array}\right)\ {P}^r\ {\left(1\mathit{\hbox{-}}P\right)}^{n\mathit{\hbox{-}}r}. $$

The rationale for using a single binomial proportion to assess the overall efficacy after administration of consecutive doses of misoprostol is that, although the probability of success varies from one dose to the other, we could say that the entire combined process was conducted with a success probability *P = P*(*Success after 2 doses*) that could be estimated by

1 $$ p=\frac{r_1+{r}_2}{n_1} $$

where *r*_1_ and *r*_2_ are the number of successes after the first and the second dose, respectively, and *n*_1_ is the number of subjects receiving the first dose; not in the formula but we also have *n*_2_ as the number of subjects receiving the second dose. When we compute the proportion of success after two doses we are assuming that there is a unique compact follow-up period during which we observe the occurrence of the event, from administering the first dose up to some predetermined time after administering the second, for example, 24 hours; the probability of the event might vary substantially during this period, but we are not interested in (or prefer to ignore) this feature [14].

Under certain circumstances ignoring this heterogeneity might not be advisable. For example, in epidemiology, if a (potential risk) factor increases the odds of a disease in men, and decreases it in women, saying that the factor does not impact the disease in the entire population might not be very useful; similarly in meta-analysis, merging individual studies with significant variability could be misleading.

As an alternative to (1), it might seem preferable to consider the following:

2 $$ \begin{array}{l}P\left( Success\  after\  2\  dose s\right)\\ {}\kern5.5em =P\left(\left( Success\  after\kern0.5em 1st\  dose\right)\  or\ \left( Success\  after\kern0.5em 2nd\  dose\mathit{\Big|} Failure\  after\ 1st\  dose\right)\right)\\ {}\kern5.75em =P\left( Success\  after\kern0.5em 1st\  dose\right)\\ {}\kern5.75em +P\left( Success\  after\kern0.5em 2nd\  dose\mathit{\Big|} Failure\  after\kern0.5em 1st\  dose\right)\ \stackrel{\wedge}{=}\frac{r_1}{n_1}+\frac{r_2}{n_2}\ \end{array} $$

as the two events are exclusive, “(*Success after 1st dose*)” and “(*Success after* 2*nd dose*|*Failure after* 1*st dose*)”. However (2) is wrong because the events come from different experiments.

Therefore, we have to approach our problem not as the sum of two exclusive events but as the (complement of the) product of two dependent events, as is done in survival analysis with the complement of the survival probability, or failure function [15, 16], that is:

3 $$ \begin{array}{l}P\left( Success\  after\ 2\  dose s\right)=1\mathit{\hbox{-}}P\left( No\  abortion\  during\  the\ 2\  first\  dose s\right)\\ {}=1\mathit{\hbox{-}}\left\{P\left( No\  abortion\  after\  1st\  dose\right)\ *\ P\left( No\  abortion\  after\ 2nd\  dose\mathit{\Big|} No\  abortion\  after\ 1st\  dose\right)\right\}\\ {}\stackrel{\wedge}{=}1\mathit{\hbox{-}}\left(1\mathit{\hbox{-}}\frac{r_1}{n_1}\right)*\left(1\mathit{\hbox{-}}\frac{r_2}{n_2}\right)\ .\ \end{array} $$

Assuming the study ends at a specific time after the last (second) dose and that there are no “censored observations” (see the next section), it can be easily proved that (1) = (3), using *n*_2_ = *n*_1_ − *r*_1_.

The standard errors for (1) and (3) are given by

4 $$ SE(p)\stackrel{\wedge}{=}\ \sqrt{\frac{p\ \left(1\mathit{\hbox{-}}p\right)}{n_1}} $$

and

5 $$ SE(p)\stackrel{\wedge}{=}\sqrt{{\left(1-p\right)}^2\left[\frac{r_1}{n_1\left({n}_1-{r}_1\right)}+\frac{r_2}{n_2\left({n}_2-{r}_2\right)}\right]} $$

respectively [15], which again can be easily proved are the same under the assumption of no censored data (*n*_2_ = *n*_1_ − *r*_1_).

Therefore, assuming there is no censored data and ignoring the heterogeneity issue mentioned above, using the binomial proportion (1) poses no problem. However, the assumption of no censored data is not realistic because in longitudinal studies censored data are usually present; in these cases *n*_2_ = *n*_1_ − *r*_1_ − *c*_1_, where *c*_1_ is the number of censored cases after the first dose and just before the second, and some *c*_2_ subjects might also be censored after administering the second dose and not available for outcome assessment at the end of the study (at a specific time after the second dose is administered).

Additionally, the problem with (3) is that in the presence of censored data *n*_1_ and *n*_2_ do not properly represent the number of subjects at risk (of medical abortion) during the two concerned periods, after the first dose (and before the second) and after the second dose (and before the end of study) respectively. We will address this in more detail in the next section.

The most common reasons for censored cases in medical abortion studies are i) surgical intervention, and ii) discontinuation or loss to follow-up. (Discontinuation because of) “End of study” is in general also a potential reason for censoring, but in these medical abortion studies “end of study” cases are usually converted into “surgical abortion” cases by design, for obvious ethical reasons [3–7, 10] and [8].

In the presence of censored cases the binomial proportion from (1) and its standard error (4) will, in general, underestimate the survival rate from (3) and will overestimate its standard error (5), respectively, and the larger the proportion of censored cases (*c*_1_/*n*_1_*or c*_2_/*n*_2_) the larger these biases.

In the next section we discuss the rationale for using a survival analysis approach and how the estimation from (3) is usually modified to account for censored data, and we will explore what drives differences between different estimations.

### The survival analysis approach

In their systematic review of more than one dose of misoprostol in medical abortion studies, Gallo et al. state that survival analysis uses more information than a binomial proportion, but they do not discuss how this additional information can or should be incorporated, and the eventual impact on the final results [9].

In a medical abortion study with multiple doses of misoprostol we usually know not only how many doses each patient may need to reach a successful abortion, but also the time (for example, hours) from treatment initiation (oral administration of mifepristone) to observing the event (expulsion of the product of conception). Therefore, we can design the survival analysis both in terms of “number of doses” or in terms of “number of hours” from treatment initiation to the event. In both cases we are using more information because the binomial proportion considers the two (or more) consecutive doses as a unique compact period (with all subjects exposed to the risk of the event throughout). A survival analysis in terms of number of hours might be difficult to interpret if we do not precisely link the results to the timing when doses were administered; on the other hand, a survival analysis in terms of number of hours makes sense under the assumption of a predetermined protocol for the timing of the successive doses.

The most relevant advantage of the survival analysis approach is that it accounts for censored data, particularly right censored data, that is, when observation ends before the event is known [15, 17], which is additional and very important information that is not used (or is misused) by the binomial proportion. In survival studies the main variable is time (or some variation) needed for a unit/subject to develop a particular characteristic or event, assuming that observation began at a properly defined time *t*_0_ for each unit. In this context the survival time for a unit might not be observed completely for different reasons (for example, loss to follow-up, end of study), and all we know is that when the subject was last seen in the study the event had not yet occurred; in these cases we say that for this subject the observation (the survival time) was right censored.

Almost always survival methods used in medical abortion studies define time as number of hours, and apply Kaplan-Meier (KM) as the estimation method ([9–11]). This method formally updates the estimation of the cumulative incidence probability each time an event (medical abortion) is observed, which in theory might occur at any time but when exactly the doses are administered.

The Life Table (LT) estimation method wastes information in comparison to the KM (LT is based on interval censored data), but it might be more appropriate when time intervals are defined a priori in terms of the timing of the successive doses or in terms of the number of doses administered, because it updates the estimation when we need it. One of the interesting features of the LT method is that assumptions about censored data can be made more flexibly and explicitly; the average number of patients at risk of the event during the interval [*t*_*j*_, *t*_*j* + 1_[⋅, ⋅ = 1, 2, and *t*_*j*_ the time when the *j*th dose is administered, is defined as

6 $$ {n}_j^{\mathit{\hbox{'}}}={n}_j-\frac{1}{2}*{c}_j $$

under the assumption that censoring is non-informative (see the following subsection) and occurs uniformly during the interval (see [18] and Section 17.2 in [13]); it can easily be seen that the average number of patients at risk during the interval from (6) can be generalized to

7 $$ {n}_j^{\mathit{\hbox{'}}}={n}_j-{k}_j*{m}_j*{c}_j $$

where *k*_*j*_ is the relative risk of the event (medical abortion) in the censored versus all those alive at the beginning of the interval, and *m*_*j*_ is the fraction of the time interval that (on average) censored cases were not exposed to the risk of the event. The corresponding estimation of the cumulative incidence probability of success is then

8 $$ P\left( Success\  after\ 2\  doses\right)\stackrel{\wedge}{=}1-\left(1-\frac{r_1}{n_1^{\mathit{\hbox{'}}}}\right)*\left(1-\frac{r_2}{n_2^{\mathit{\hbox{'}}}}\right) $$

for *n*_*j*_^′^ from (6) (or (7)), with (8) now properly accounting for censored cases; the corresponding standard error is obtained from (5) just replacing *n*_*j*_ by *n*_*j*_^′^,

9 $$ SE(p)\stackrel{\wedge}{=}\sqrt{{\left(1-p\right)}^2\left[\frac{r_1}{n_1^{\mathit{\hbox{'}}}\left({n}_1^{\mathit{\hbox{'}}}-{r}_1\right)}+\frac{r_2}{n_2^{\mathit{\hbox{'}}}\left({n}_2^{\mathit{\hbox{'}}} - {r}_2\right)}\right]}\ . $$

Observe that in the case of just one dose of misoprostol (max j = 1), and under the assumption of no censored data, (8) and (9) become exactly the point estimate and the corresponding standard error of the binomial proportion, (1) and (4), respectively. Also note that both the KM and the LT methods consider the competing events (see definition in the following subsection) of surgical abortions as censored data and are therefore included in *c*_*j*_, *j* = 1,2.

#### Accounting for competing risks

In any longitudinal study censored data are observations that are interrupted for some reason(s); the events generating these interruptions may or may not influence the probability of occurrence of the event of interest. If censoring (of our main event observation) is caused by an alternative event that modifies the probability of observing the main event, the censoring is called “informative”; otherwise, the censoring is called “non-informative” (for example, as was assumed for [6] above).

A competing risk is defined as any event that precludes (or modifies the probability of) the onset of the event of interest [16, 19]; therefore, a competing risk is an event that generates a particular type of censored data, or “informative” censored data. For example, surgical abortion is a “competing risk” for our main event medical abortion because the occurrence of the first prevents (definitely modifies!) the occurrence of the second; alternatively we can say that we have two different and competing (mutually exclusive) ways of induced abortion, “medical” and “surgical”.

It is evident that a “competing risk” can have different levels according to the degree to which it influences the probability of the occurrence of our main event. We can also have, of course, more than two competing risks, and potentially they can all compete with each other at different levels.

The classical KM and LT estimations do not account for competing risks because they always assume non-informative censoring; their results can be interpreted as the survival probabilities under the assumption that competing risks are non-existent (or have been eliminated), because considering competing risks as non-informative censoring is, in practice, the closest we can get to ignoring them. This might be a reasonable approach if we want to assess (extrapolate) the performance of medical abortion in a (to a counter-factual) context where surgical abortion is non-existent; however, in a context where both medical and surgical abortion are and will be available, the results from the classical KM and LT methods are not appropriate because the survival rate is underestimated (or the cumulative incidence probability is overestimated).

If there are no competing risks (that is, surgical abortion does not exist), we can express the distribution function (or cumulative incidence probability) of medical abortion (8) above as

10 $$ P\left( Success\  after\ 2\  doses\right)\stackrel{\wedge}{=}{\displaystyle \sum_{j=1}^2S\left({t}_{j\mathit{\hbox{-}}1}\right)*\frac{r_j}{n_j^{\mathit{\hbox{'}}}}=S\left({t}_0\right)*\frac{r_1}{n_1^{\mathit{\hbox{'}}}}+S\left({t}_1\right)*\frac{r_2}{n_2^{\mathit{\hbox{'}}}}} $$

where *S*(*t*_*j*_) is the probability of survival up to *t*_*j*_ and *S*(*t*_0_) = 1; for example, *S*(*t*_1_) * *r*_2_/*n*_2_^′^ is the probability of having a medical abortion after the second dose (and before the planned end of study) given that the patient did not have a medical abortion after the first dose (and before the second).

When *k*(*k* = 1 *to K*) competing risks are present, [10] can be modified to

11 $$ P\left( Success\  after\kern0.5em 2\  doses\  of\  event\ k\right)\stackrel{\wedge}{=}{\displaystyle \sum_{j=1}^2{S}_k\left({t}_{j\mathit{\hbox{-}} 1}\right)*\frac{r_{k,j}}{n_j^{\mathit{\hbox{'}}}}} $$

which is equivalent to (10), or to

12 $$ P\left( Success\  after\kern0.5em 2\kern0.5em  doses\  of\  event\ k\right)=CRC{I}_k(2)\stackrel{\wedge}{=}{\displaystyle \sum_{j=1}^2S\left({t}_{j\mathit{\hbox{-}}1}\right)*\frac{r_{k,j}}{n_j^{\mathit{\hbox{'}}}}} $$

with *S*_*k*_(*t*_*j*−1_) the probability of survival just to the *k*^*th*^ event, and *S*(*t*_*j*−1_) the probability of survival to all *K* events; the competing risk cumulative incidence rate *CRCI*_*k*_(2) from (12) is a sub-distribution function and is known as the “*crude* (cumulative) incidence function”, “*k*-specific risk” or “*k*-specific probability of failure” [20, 21]. Note that we keep on including the actuarial assumptions in (10) to (12) from the Life-Table approach, but as far as we know, all packages implementing the *CRCI*_*k*_(*j*) rates use the KM approach assuming that the exact timing of events is known.

The probability of the *k*^*th*^ event in the *j*^*th*^ interval counts only occurrences of the *k*^*th*^ event both in (11) and (12); but in (11) survival considers all other competing events as censored (that is, the subject has to survive only to the *k*^*th*^ event) while in (12) survival includes all competing events. The standard KM and LT approaches use (11), which overestimates (12). In fact, it can be proved in general that $CRC{I}_k(2)\le {\displaystyle {\sum}_{j=1}^2{S}_k\left({t}_{j-1}\right)*\frac{r_{k,j}}{n_j^{\prime }}\le \left(1-S(2)\right)}\kern0.5em \mathrm{and}\kern0.5em {\displaystyle {\sum}_{k=1}^KCRC{I}_k(2)=\left(1-S(2)\right)}$, with *S*(2) the probability of having no abortion at all after taking two misoprostol doses [19, 21].

The rationale of (12) is obviously that to have a particular type of abortion (medical) during the *j*^*th*^ interval, patients need to survive up to the beginning of this interval to both medical and surgical abortion (not only to medical); see, e.g., [19] and [21].

There are several options for the standard error of (12) [21]; the one usually recommended and implemented in the Stata command *stcompet* [19] and in the *cmprsk* R package [22] is

13 $$ \begin{array}{c}\ se\left(CRC{I}_k(2)\right)\ \stackrel{\wedge}{=}\left\{{\left[CRC{I}_k(2)-CRC{I}_k(1)\right]}^2\ \frac{r_{{}_1}}{n_1\left({n}_{{}_1}\mathit{\hbox{-}}{r}_1\right)} + \frac{n_1\mathit{\hbox{-}}{r}_{k{,}_1}}{n_1}*\frac{r_{k{,}_1}}{n_{{}_1}^2}\ \right.\\ {}+{\left[S(1)\right]}^2\ \frac{n_2\mathit{\hbox{-}}{r}_{k,2}}{n_2}*\frac{r_{k,2}}{n_2^2}{\left.-2*\left\{\left[CRC{I}_k(2)\mathit{\hbox{-}}CRC{I}_k(1)\right]*\frac{r_{k,1}}{n_1^2}\right\}\right\}}^{\raisebox{1ex}{$1$}\!\left/ \!\raisebox{-1ex}{$2$}\right.}\ \end{array} $$

with ∑_*k* = 1_^*K*^*r*_*k*,*j*_ = *r*_*j*_; an appropriate function of (13) for computing the confidence interval is given in [19].

It should be noted that in the case of just one dose of misoprostol (max j = 1), the CRCI estimate (12) becomes the binomial proportion in (1), with the corresponding standard errors being also equal, (13) and (4) respectively. It should also be noted that independent of the number of doses, the same happens when non-informative censored data are absent (*c*_1_ = *c*_2_ = 0).

In Table 1 we compare the point estimates and their standard errors when using the binomial (bin), the Kaplan-Meier (KM), the Life Table (LT), and the competing risk (CRCI) approaches, under 18 scenarios for the rates of the main event (medical abortion), the competing event (surgical abortion), and censored data (all other discontinued/loss-to-follow-up cases); in all scenarios the censored data after the second dose have been mostly (re)converted into the competing event “surgical abortion” as per the usual design of these studies (see details of Table 1 in Methods). For the survival approaches (KM, LT, and CRCI) all events were assumed to occur exactly at times (doses) 1 or 2; if we had instead used other (more realistic) timings the results would be slightly different, but the trends observed would remain the same.

It can be seen that the binomial proportion (bin) underestimates the success rate after two doses of misoprostol, mainly when using the LTapproach which includes the actuarial assumptions (columns {9}–{11}); the KM and LT approaches are appropriate if we want to extrapolate to a population/situation where the competing event (surgical abortion) does not exist. The underestimation of bin with respect to CRCI is much smaller (columns {9} and {12}), the CRCI rates being appropriate when we want/expect medical and surgical abortion to coexist in our population.

From Table 1 we see that the higher the censoring rate the higher the under-estimation by the bin approach. We also see that the lower the rate of the main event with the second dose with respect to that of the first dose, the smaller the under-estimation of the bin approach; in these cases the combined rate would be mostly dominated by the rate with only the first dose, meaning that the second dose would be close to irrelevant and the binomial approach would then be close to being adequate. Finally we see that the higher the success rate with the first dose, the smaller the underestimation of the combined rate using the bin approach with respect to the combined rate estimations from the survival approaches.

The comparison of standard errors (columns {14}–{18} on Table 1) shows that the bin approach has consistently larger standard errors than the survival approaches; that is, it underestimates the precision of the success rate estimation after two doses, particularly in comparison to the LT approach (columns {14} and {16}). As when comparing point estimations, the smaller the success rate with the second dose (in comparison to the rate with the first dose) the smaller the underestimation of precision of the bin approach with respect to the survival approaches; also, in general the higher the rates of competing events and censoring, the higher the underestimation of precision of the bin approach with respect to the survival approaches. In contrast to the comparison of point estimations, the higher the rate of the main event with the first dose, the higher the underestimation of precision of the bin approach compared to the survival approaches. Although the precision of the bin approach increases with higher rates of the main event with the first dose, we see that the corresponding precisions for the survival approaches increase faster.

In terms of point estimations’ biases (underestimation) of the bin approach the worst scenario is the sixth, corresponding to a low (70 %) rate of the main event with the first dose, a high (66 %) rate of the main event with the second dose, and high rates (20 % with the first dose) of competing events and censoring. In terms of precision biases (underestimation) of the bin approach the worst scenario is the 18th, corresponding to a high (90 %) rate of main event with the first dose, a high (85 %) rate of the main event with the second dose, and high rates (20 % with the first dose) of competing events and censoring.

## Discussion

The binomial proportion approach is still considered the gold standard for assessing medical abortion success when using multiple doses of misoprostol; we estimate from the literature reviewed that in at least 80 % of the relevant studies the binomial proportion approach is being used. We think the main reason is its seductive simplicity. Many researchers are probably unaware of its limitations, and the main purpose of this study is to make these explicit.

The survival analysis approach, in any of the three versions considered here, is adequate because it accounts for non-informative censoring in general and, eventually also for competing risk events (informative censoring). The superiority of the survival approach does not depend on the length of the periods between doses (days or hours), but mainly on the number of censored observations between consecutive doses; the larger the number of censored observations the larger the bias introduced by the binomial approach.

Medical abortion studies using misoprostol do not usually have censored observations owing to loss to follow-up (including “end of study”), because they are designed so that all patients achieve success (complete abortion) one way or the other, before the end of the study. However, a non-negligible number of patients might have their observations censored because of experiencing the competing risk event “surgical abortion”, which is not accounted for by the binomial approach.

WHO guidelines indicate that following administration of misoprostol (using the combined regimen or mifepristone and misoprostol for women with pregnancies of gestational age up to 9 weeks) up to 90 % of women will expel the products of conception (page 45, [1]). If we assume that for gestational ages above 9 weeks the success rate (with the first dose of misoprostol) is in general below 90 %, then the scenarios in Table 1 are relevant from a clinical point of view.

When the time period between consecutive doses has not been measured precisely, we could still use the survival approach, in this case not in terms of time (days or hours) but in terms of number of doses; this will keep the advantage of the survival approaches of accounting for censored observations.

Because a binomial proportion does not account for the fact that different individuals are exposed to different time periods to the event of interest, as an alternative we might be tempted to use a Poisson rates’ approach to account for this exposure heterogeneity. However, Poisson rates (usually) assume that the probability of the event is the same across time, which is generally not the case in medical abortion studies using multiple doses of misoprostol.

The “intermediate outcome” approach, using for example “principal stratification” might initially seem an attractive alternative way to tackle the problem, because it could be argued that surgical abortion is a “truncation by death” intermediate outcome for medical abortion [23]. However, the concept of intermediate outcomes requires the intermediate event to be in the causal path between intervention and the outcome, which is not the case of surgical abortion (between the “nurses” intervention and the outcome “medical” abortion). Surgical abortion precludes medical abortion, but it is not a “cause” of medical abortion; it is not possible to treat surgical abortion independent of medical abortion (in the sense of one being the potential cause of the other), because they are just two (alternative and competing) ways of reaching the same outcome/goal.

## Conclusions

From the analysis and results of this paper we conclude and recommend that binomial proportions should always be replaced by survival rates when assessing/comparing success rates of medical abortion with multiple doses of misoprostol. The main reason is that binomial proportions do not account for censored data or for competing risks, and as a consequence:

• Binomial proportions tend to underestimate the real success rate. The smaller the medical abortion rate of success (closer to 50 % from above) and the larger the rate of the surgical abortion rate (closer to 50 % from below), the larger the underestimation in comparison to the survival approaches (Kaplan-Meier, Life Table, and Cumulative Incidence); and

• Binomial proportions tend to overestimate the real standard error. This means that when binomial proportions are used there is a tendency to miss statistically significant differences between rates of success because the corresponding standard errors are being overestimated. The larger the medical abortion rate of success (closer to 100 %) and the larger the rate of the competing surgical abortion event (closer to 50 % from below), the larger the overestimation of standard error.

If we were interested in estimating the success rate of medical abortion after two or more doses *under the assumption (counter-factual) that surgical abortion does not exist (or it has been eliminated)*, then the Kaplan-Meier and Life Table approaches are suggested as appropriate because they consider the competing event (surgical abortion) as non-informative censoring [20].

On the other hand, if we want to estimate the success rate of medical abortion in the presence of competing risks, in this case surgical abortion, then the use of the Competing Risk Cumulative Incidence (CRCI) survival approach is indicated [19, 20]; when using this approach it is important to present the rates for each of the competing events (in this case “medical” and “surgical” abortion), as the interpretation of the rate of the main event may depend on the rate(s) of the other competing event(s) [20].

In some cases the broad conclusions might remain the same independent of the approach used, but there is no advantage in choosing an option that is consistently biased with respect to more appropriate methods which are now widely available in standard statistical packages.

Our recommendations can be extended to other sexual and reproductive health studies where success is assessed after multiple rounds/administrations of the (experimental) treatment. For example, in assisted reproductive technology (ART) for fertility treatment success has traditionally been reported on a per-cycle basis using binomial proportions; survival techniques are increasingly used to estimate the cumulative rate of achieving an ongoing pregnancy (or live pregnancy) after successive cycles of in vitro fertilization or other ART techniques and some adjustments for informative censoring have been included [24, 25], but more formal competing risks approaches could also be applied.

## Abbreviations

ART: assisted reproductive technology
CRCI: Competing Risk Cumulative Incidence rate
KM: Kaplan-Meier
LT: Life Table
WHO: World Health Organization
